# Association of pre-surgery to pre-radiotherapy lymphocyte counts ratio with disease-free survival in rectal cancer patients receiving neoadjuvant concurrent chemoradiotherapy

**DOI:** 10.1186/s12957-019-1747-9

**Published:** 2019-11-30

**Authors:** Hongen Xu, Guangxian You, Minjun Zhang, Tao Song, Haibo Zhang, Jia Yang, Yongshi Jia, Jianming Tang, Xiaodong Liang

**Affiliations:** 1 Department of Radiation Oncology, Cancer Center, Zhejiang Provincial People’s Hospital, People’s Hospital of Hangzhou Medical College, No. 158, Shangtang Road, Xiacheng District, Hangzhou, 310014 China; 2 Department of Radiation Oncology, Taizhou Cancer Hospital, Taizhou, 317502 China; 3 Graduate Department, Bengbu Medical College, No. 2600 Donghai Avenue, Bengbu, 233000 China

**Keywords:** Rectal cancer, Lymphocyte counts, Disease-free survival, Chemoradiotherapy

## Abstract

**Background:**

Colorectal cancer is the fourth most common cancer globally and neoadjuvant concurrent chemoradiotherapy (nCRT) and surgery are the standard treatments for locally advanced colorectal carcinoma. This study investigated the association between dynamic changes in absolute lymphocyte counts (ALCs) and disease-free survival (DFS) in rectal cancer patients receiving nCRT and identified factors associated with these changes.

**Methods:**

We retrospectively examined 34 patients with locally advanced rectal cancer who received nCRT followed by surgery and adjuvant chemotherapy. The association between ALCs and DFS and that between ALCs and downstaging were analyzed and potential clinical- and treatment-related factors related to dynamic changes in ALCs were subsequently evaluated. The patient eligibility criteria were as follows: pathologically confirmed rectal adenocarcinoma, clinical stages II–III, ≥ 18 years of age, and so on. Pre-RTL was defined as ALCs obtained before the initiation of nCRT and pre-SL was defined as ALCs obtained before surgery. We measured pre-SL to pre-RTL ratio (pre-SLR), DFS, and ALCs.

**Results:**

The median ALC declined significantly during nCRT. A lower pre-SLR was associated with poorer DFS with statistical significance in Kaplan–Meier (*p* = 0.007), univariate regression (hazard ratio [HR] = 6.287, 95% confidence interval [CI] 1.374–28.781, *p* = 0.018), and multivariable regression (HR = 7.347, 95% CI 1.595–33.850, *p* = 0.011) analyses. Neither patient characteristics nor treatment-related factors were related to downstaging. The pelvic bone marrow (PBM) volume receiving at least 30 Gy (V30) was significantly associated with pre-SLR in the univariate (HR = 5.760, 95% CI 1.317–25.187, *p* = 0.020) and multivariable (HR = 5.760, 95% CI 1.317–25.187, *p* = 0.020) regression analyses.

**Limitations:**

Our study had several limitations. The sample size was small and the study was performed in a selected population, which may limit the generalization of the findings.

**Conclusions:**

Radiotherapy had a profound impact on the change in ALCs. A lower pre-SLR was significantly associated with poorer DFS in rectal cancer patients receiving nCRT. The V30 of PBM was a predictor of pre-SLR.

## Background

The management of local advanced colorectal cancer is timely. Colorectal cancer is the fourth most common cancer globally [1]. Although significant improvements in the treatment of rectal cancer have recently been made, the overall 5-year survival rate for the whole population is dismal, at less than 60%. Compared with adjuvant chemoradiotherapy, neoadjuvant concurrent chemoradiotherapy (nCRT) improves the local control rate and decreases toxicities. nCRT has also been shown to increase the rate of sphincter preservation and improve quality of life [2, 3]. nCRT and surgery are, therefore, the standard treatments for locally advanced colorectal carcinoma.

A series of studies has shown that the host immune system is significantly associated with the prognosis of patients with colorectal cancer. Tumor-infiltrating lymphocytes [4, 5] are associated with the prognosis in colorectal cancer. However, nCRT may have a detrimental effect on host immune function. Among all immune factors, lymphocytes play a key role in host immune function. Circulating lymphopenia has been shown to be a prognostic factor in non-small-cell lung cancer, esophageal cancer, pancreatic carcinoma, and other solid cancers [6]. In rectal cancer, lymphocyte count is both a predictor and a prognostic factor. A high ratio of lymphocytes in white blood cells before treatment is associated with not only higher pathologic complete remission but also better disease-free survival (DFS) and overall survival (OS) in rectal cancer [7]. Similarly, a better pathologic complete response rate was achieved in patients with higher sustained lymphocyte counts at 4 weeks during nCRT [8]. In addition, lymphopenia is associated with prognosis in patients with advanced colorectal cancer receiving chemotherapy [9].

However, the association between DFS and dynamic changes in ALCs is not well documented in the literature. The present study aimed to explore whether DFS is associated with circulating ALCs before chemoradiotherapy (pre-RTL), during nCRT, and before surgery (pre-SL) in patients with locally advanced rectal cancer and to explore the potential clinical and treatment-related factors associated with lymphocyte counts.

## Materials and methods

### Patient selection

This study was reviewed and approved by the medical ethics committee of Zhejiang Provincial People’s Hospital. Informed consent was waived by the board due to the retrospective design of this study. The patient eligibility criteria were as follows: pathologically confirmed rectal adenocarcinoma, clinical stages II–III, ≥ 18 years of age, Eastern Cooperative Oncology Group (ECOG) performance status of 0–1, baseline complete blood counts and carcino-embryonic antigen (CEA) data available, received nCRT followed by surgery-at our institution, total mesorectal excision (TME) performed with R_0_ resection, no history of previous radiotherapy or chemotherapy, no history of previous or coexisting cancer, and at least three weekly complete blood counts documented during nCRT. ALCs were obtained from complete blood count tests. ALCs obtained before the initiation of nCRT (within 1 week) were defined as pre-RTL, while ALCs obtained before surgery (within 1 week) were defined as pre-SL. The pre-SLR was defined as the ratio of pre-SL to pre-RTL. The lowest ALC was considered as the lymphocyte nadir during nCRT (W1 to W5). Lymphocyte toxicity was evaluated according to the National Cancer Institute’s Common Terminology Criteria for Adverse Events (CTCAE) version 4.0. Downstaging was defined as pathologically staged T0–2 N0 after surgery (ypT0–2 N0).

Patients were followed up every 3 months for 2 years after surgery, every 6 months in the third to fifth year, and then on a yearly basis. Local recurrence was considered as a recurrence within the pelvis (below the fifth lumbar spine L5). New lesions beyond the pelvis were considered distal metastasis. DFS was calculated from the start date of CRT to the date of first documented radiographic failure or death from any cause.

### Evaluation of the PBM dose

The external contour of all the bones within the pelvis was contoured as pelvic bone marrow (PBM) on computed tomography (CT) simulation (5-mm slice thickness), from the bottom of the fourth lumbar vertebra to the bottom of the ischial tuberosities. The dose-volume histogram (DVH) parameters of the PBM were generated using a Pinnacle Treatment Planning System. The V5, V10, V20, V30, and V40 parameters were calculated as the percentages of volumes receiving at least 5, 10, 20, 30, and 40 Gray (Gy), respectively. The D_mean_ was calculated as the mean dose to the PBM.

### Statistical analysis

Friedman tests were used to compare the ALCs at different time points during nCRT. DFS was calculated (the Kaplan–Meier method) and compared (log-rank tests) between groups. The association between clinical data and downstaging was evaluated by Fisher’s exact tests. The associations between potential prognostic factors and DFS were evaluated by univariate and multivariate analyses using a Cox regression model. We further analyzed the association between clinical data and the pre-SLR level in univariate and multivariate analyses. The multivariate analysis was performed with logistic regression backward stepwise methods, including all of the variables used in univariate analysis. The variables were considered as dichotomous variables. Lymphocyte nadir was dichotomized at 0.5 × 10^9^ cells/L. All other continuous variables were dichotomized at the median level. All statistical analyses were performed using IBM SPSS Statistics for Windows, version 25.0 (IBM Corp., Armonk, NY). Significant differences were defined as two-sided p-values of less than 0.05.

## Results

### Patient and treatment characteristics

A total of 41 patients with pathologically diagnosed rectal cancer were treated in our institution with nCRT between January 2013 and December 2017. Seven patients were excluded from the study for other cancers (two patients), resectable liver metastases (four patients), and incomplete data (one patient). Thirty-four patients with complete clinical data were finally included in the current study. Their characteristics are shown in Table 1. Standard TME was performed in all patients with negative margin (R_0_ resection).

**Table 1:** Patient characteristics

Characteristics	
Number of patients	34
Median age (range)	59 (29–79)
Sex	
Male	25 (73.5%)
Female	9 (26.5%)
Clinical T stage	
T1–2	1 (2.9%)
T3–4	33 (97.1%)
Clinical N stage	
N−	3 (8.8%)
N+	31 (91.2%)
CEA (ng/mL)	
< 5.0	17 (50%)
≥ 5.0	17 (50%)

*N*, lymph node; *N+*, lymph node positive; *N−*, lymph node negative; *CEA*, carcino-embryonic antigen

### nCRT

Patients were required to undergo CT simulation with contrast. The clinical target volume (CTV) included the gross tumor with a margin of 2–3 cm, mesorectum, presacral lymph nodes, and internal iliac lymph nodes. The CTV_boost_ comprised the gross tumor with a 2–3 cm margin. The planning target volume (PTV) received a dose of 45 Gy and the PTV_boost_ received a dose of 50 Gy in 25 fractions simultaneously, using intensity-modulated radiotherapy (IMRT) technique. The median PTV was 1617 (range, 859 to 2166) cc. Treatment plans were generated on a Pinnacle Treatment Planning System (v 9.8), using 6-MV or 10-MV photons. All patients were treated on a Siemens Oncor Impression Plus accelerator. Capecitabine was administered at a dose of 1650 mg/m^2^/day during nCRT.

### Surgery and postoperative chemotherapy

Surgery was performed 6–10 weeks after the completion date of nCRT. After the operation, postoperative chemotherapy was administered. The postoperative chemotherapy comprised 5–8 (median 7) cycles of FOLFOX (a regimen of 5-FU, leucovorin, and oxaliplatin every 2 weeks), 4–6 (median 5) cycles of XELOX (a regimen of capecitabine and oxaliplatin every 3 weeks), or 4–6 (median 5) cycles of capecitabine monotherapy (every 3 weeks).

### ALCs during treatment

**Fig. 1:**
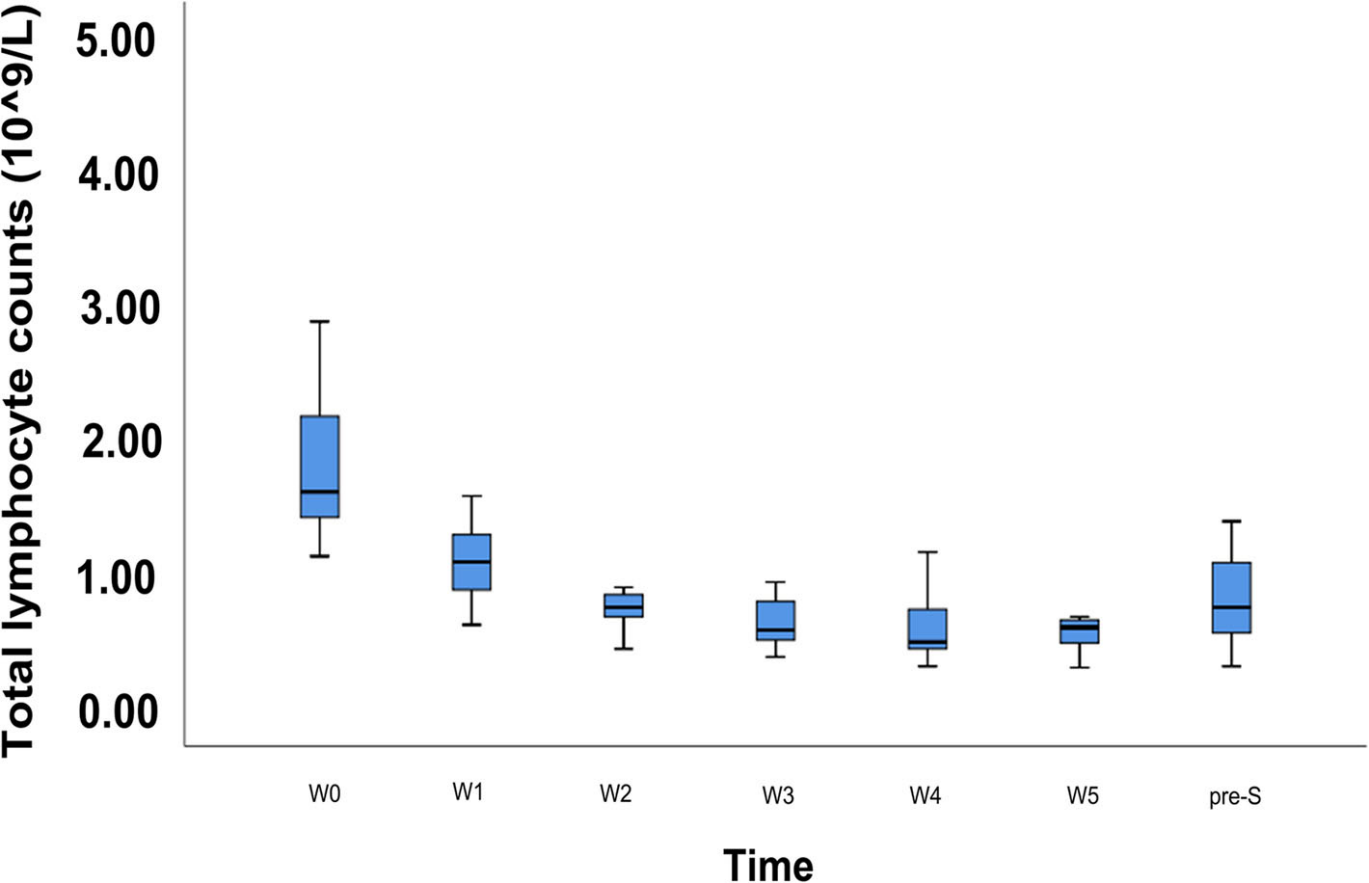
Absolute lymphocyte counts (ALCs) changed over time. Significant differences were found between ALCs at W0 and W1 (*p* = 0.041), W0 and W2 (*p* < 0.001), W0 and W3 (*p* < 0.001), W0 and W4 (*p* < 0.001), and W0 and W5 (*p* < 0.001). W0: pre-RT; W1–5: week 1 to week 5 during CRT; pre-S: before operation

Prior to treatment (defined as W0), 24 patients (70.59%) had normal ALC, 10 patients (29.41%) experienced grade 1 lymphopenia, and no patient had grade 2–4 lymphopenia. Before operation, two patients (5.88%) had normal ALC, six patients (17.65%) had grade 1 lymphopenia, 22 patients (64.71%) had grade 2 lymphopenia, four patients (11.76%) had grade 3 lymphopenia, and no patient had grade 4 lymphopenia. The median ALC at baseline (W0) was 1.62 (range, 1.14–4.36) × 10^9^ cells/L. The median ALCs declined to 1.10 (range, 0.63–2.02), 0.76 (range, 0.45–1.87), 0.59 (range, 0.39–1.36), 0.50 (range, 0.32–1.27), and 0.61 (range, 0.31–1.08) × 10^9^ cells/L from week 1 (W1) to week 5 (W5) during CRT, respectively. The median pre-SL was 0.76 (range, 0.32–2.32) × 10^9^ cells/L. Significant differences were found between ALCs at W0 and W1 (*p* = 0.041), W0 and W2 (*p* < 0.001), W0 and W3 (*p* < 0.001), W0 and W4 (*p* < 0.001), W0 and W5 (*p* < 0.001), W1 and W2 (*p* = 0.020), and W5 and pre-SL (*p* = 0.010). However, no significant difference was detected between ALCs at W2 and W3 (*p* = 0.056), W3 and W4 (*p* = 0.474), and W4 and W5 (*p* = 0.973, detailed in Fig. 1).

### Clinical and treatment characteristics associated with DFS

**Fig. 2:**
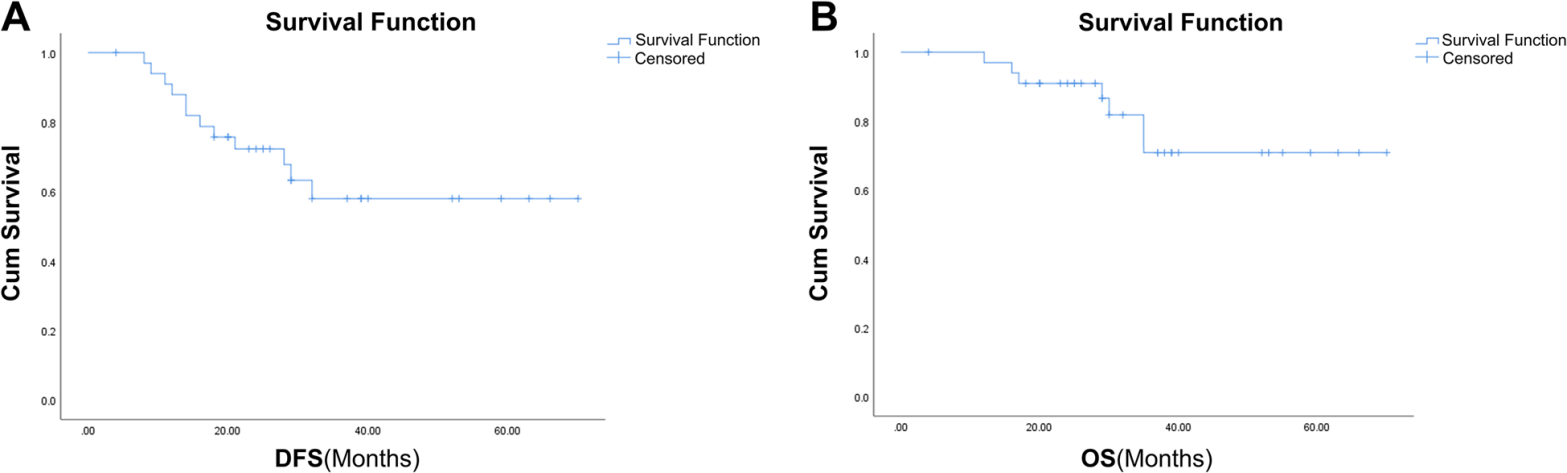
Disease-free survival (DFS) and overall survival (OS) in the whole patient cohort

**Fig. 3:**
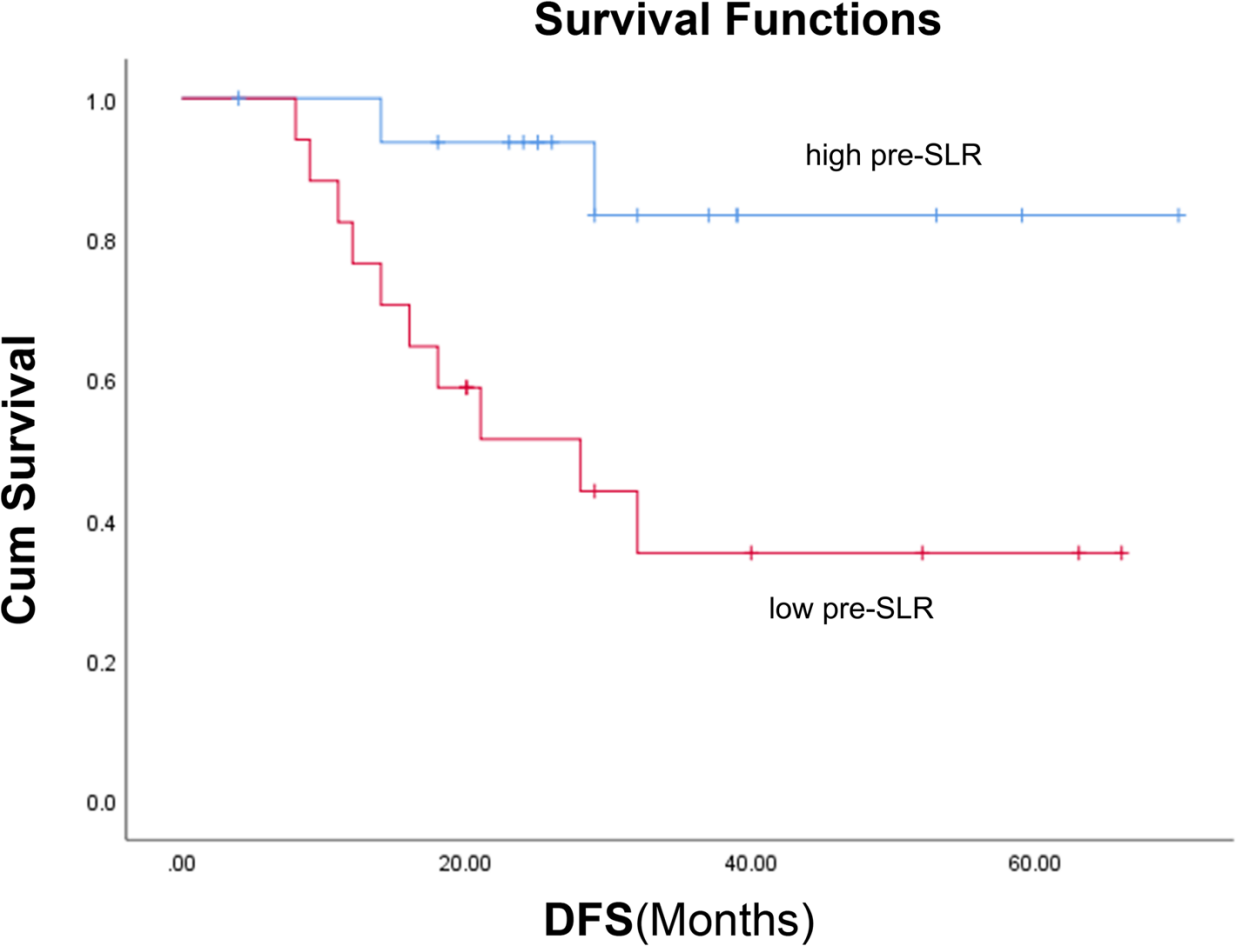
DFS curves stratified by pre-SLR level

The DFS ranged from 4 to 70 months and the median DFS of all patients had not been reached through the last follow-up date of December 31, 2018. Twenty-seven patients were alive while 7 patients died of disease progression. The median follow-up time of the surviving patients was 32 months. Twelve patients had progressive disease. Ten of them had distant metastases, one had a local recurrence, and one had both. Pre-SLR was the only prognostic factor (Table 2) with significant associations with DFS in both univariate (HR = 6.287; 95% confidence interval [CI], 1.374–28.781; *p* = 0.018) and multivariate analyses (HR = 7.347; 95% CI, 1.595–33.850; *p* = 0.011). No other factor was significantly associated with DFS, including age, sex, CEA, downstage, positive lymph node, pre-RTL, pre-SL, lymphocyte nadir, and PTV. Figure 2 shows the OS and DFS curves of the whole cohort and Fig. 3 shows Kaplan–Meier DFS curves stratified by pre-SLR. The median DFS was 28 months in patients with a low pre-SLR and the median DFS had not been reached in patients with a high pre-SLR (*p* = 0.007).

**Table 2:** Univariate and multivariate analysis for variables associated with DFS

Clinical factors		Univariate analysis			Multivariable analysis		
		HR	95% CI	*p*	HR	95% CI	*p*
Age	(≥ 59 vs< 59)	0.935	0.338–3.255	1.048			
Sex	(F vs M)	0.428	0.093–1.980	0.278			
CEA	(≥ 5.0 vs < 5.0)	0.980	0.315–3.049	0.972	–		
Downstage	(n vs y)	2.104	0.459–9.653	0.338			
N status	(N+ vs N−)	1.924	0.610–6.07	0.264			
Pre-SL	(L vs H)	3.363	0.987–13.656	0.052			
Pre-RTL	(L vs H)	0.433	0.095–1.983	0.281			
Lnadir	(L vs H)	1.804	0.542–6.003	0.336			
Pre-SLR	(L vs H)	6.287	1.374–28.781	0.018	7.347	1.595–33.850	0.011

*HR*, hazard ratio; *CI*, confidence interval; *F*, female; *M*, male; *CEA*, carcino-embryonic antigen; *N*, lymph node; *N+*, lymph node positive; *N−*, lymph node negative; *pre-SL*, absolute lymphocyte counts before surgery; *pre-RTL*, absolute lymphocyte counts before concurrent chemoradiotherapy; *Lnadir*, the lowest absolute lymphocyte count during concurrent chemoradiotherapy; *n*, no; *y*, yes; *L*, low; *H*, high

### Patient characteristics and treatment factors associated with pre-SLR

Table 3 shows that PBM V30 (median 47.18%; range 28.01–59.14%) was the only predictive factor for pre-SLR in both univariate and multivariate regression analyses. PBM V20, PBM V40, PBM D_mean_, and PTV were not significantly associated with pre-SLR in univariate analysis or multivariate analysis (Table 3).

**Table 3:** Univariate and multivariate analysis for variables associated with pre-SLR

Clinical factors		Univariate analysis			Multivariable analysis		
		HR	95% CI	p	HR	95% CI	p
Age	(≤ 60 vs > 60)	1.257	0.333–4.742	0.735			
Sex	(F vs M)	2.062	0.492–8.654	0.322			
N status	(N+ vs N−)	1.250	0.299–5.230	0.760			
CEA	(> 5.0 vs ≤ 5.0)	0.606	0.158–2.319	0.464			
Pre-RTL	(L vs H)	8.000	1.686–37.981	0.009			
V5	(L vs H)	4.469	1.054–18.938	0.042			
V10	(L vs H)	2.041	0.521–7.999	0.306			
V20	(L vs H)	3.361	0.823–13.722	0.091			
V30	(L vs H)	5.760	1.317–25.187	0.020	5.760	1.317–25.187	0.020
V40	(L vs H)	3.361	0.823–13.722	0.091			
Dmean	(L vs H)	3.361	0.823–13.722	0.091			

*HR*, hazard ratio; *CI*, confidence interval; *F*, female; *M*, male; *CEA*, carcino-embryonic antigen; *N*, lymph node; *N+*, lymph node positive; *N−*, lymph node negtive; *pre-SL*, absolute lymphocyte counts before surgery; *pre-RTL*, absolute lymphocyte counts before concurrent chemoradiotherapy; *n*, no; *y*, yes; *L*, low; *H*, high

### Patient characteristics and treatment-related factors associated with downstaging

Neither patient characteristics nor treatment-related factors were related to downstaging, including pre-RTL, ALC nadir, pre-SLR, sex, age, CEA, and PTV. Furthermore, none of these factors was related to positive lymph node status.

## Discussion

It is widely accepted that the host anti-tumor immune response is important in patients with cancer. The association between circulating lymphocyte count and DFS has not been well documented in locally advanced rectal cancer. In the present study, we reported that circulating ALCs declined significantly during nCRT and that pre-SLR was the only prognostic factor for DFS in rectal cancer. The V30 of the pelvic bone marrow was further found to be the only predictive factor inversely associated with a high pre-SLR.

To our knowledge, this is the first study to report the association between pre-SLR and DFS in patients with locally advanced rectal cancer receiving nCRT followed by TME. Heo and coauthors reported that a sustained lymphocyte ratio in the fourth week of preoperative CRT was a predictor for pathologic complete response in rectal cancer [8]. However, survival data were not available in that study. Another study reported the initial lymphocyte ratio to be a predictor of pathologic complete response in rectal cancer. In addition, lymphocyte ratio was also a predictor of DFS [7]. Treatment-related lymphopenia has been verified to be associated with prognosis in other cancers. Tang et al. found that lower ALC after definitive radiotherapy was associated with shorter OS and event-free survival in non-small lung cancer [10]. Cho and coauthors verified that lymphocyte nadir was a prognostic factor in limited-stage small-cell lung cancer [11]. Neither pre-RTL nor pre-SL was associated with DFS in the present study. It is possible that the patient number was too low to observe an association between ALCs and DFS. However, a high pre-SLR was a useful predictor for better DFS. Therefore, pre-SLR may be a better prognostic factor than pre-RTL and pre-SL because it considers both pre-CRT and post-CRT ALCs.

We also found that circulating ALCs declined drastically during nCRT, a finding in accordance with previous studies and has been suggested to be due to lymphocyte sensitivity to radiotherapy [7, 8, 10–12]. This finding further indicates the importance of sustaining lymphocyte number during CRT in routine clinical practice. Circulating lymphocyte numbers are easily affected by various factors. However, none of the clinical characteristics in the present study, including age, sex, lymph node status, and CEA, were associated with the pre-SLR.

We further found that the V30 of the PBM significantly affected the pre-SLR. The irradiation dose to the PBM is reported to be significantly associated with hematologic toxicity in rectal cancer [13, 14]. Similar results were reported in cervical and anal cancer [15–19]. However, the influence of radiotherapy dose to the PBM on lymphocyte counts was not described. Our results indicate that the irradiation dose to the PBM may be related to lymphocyte counts. Limiting the irradiation dose to the PBM might help to preserve ALCs. We will initiate a clinical trial to investigate whether bone marrow–sparing IMRT can help rectal cancer patients to sustain higher pre-SLR levels.

Peripheral lymphocyte counts and lymphocyte subtypes were also predictors of favorable response after nCRT in rectal cancer [7, 8]. However, no clinical factors were associated with downstage or positive lymph node in the present study, including pre-RTL, ALC nadir, pre-SLR, sex, age, CEA, and PTV volume. Many predictive factors for pathological responses have been reported in rectal cancer, including CEA, distance from the anal verge [20], p21, and other factors [21]. However, currently, there is no widely accepted predictive factor. Therefore, it is reasonable that our results were not concordant with those of previous studies regarding the predictive factors for pathological downstaging.

There are several limitations in the present study. First, bias is possible because it is a small single-center retrospective study. Our results should be verified in larger prospective studies. Second, lymphocyte subpopulations were not examined and we did not know the lymphocyte subtypes associated with DFS. The changes in lymphocyte subpopulation should be assessed in future studies. Third, we used the entire pelvic bone to represent the pelvic bone marrow and more precise methods to evaluate active bone marrow such as positron emission tomography-computed tomography (PET-CT) were not considered. However, pelvic bone delineation was easy to perform, had a low cost and was time-saving.

## Conclusion

The present study suggests that pre-SLR may be a useful prognostic factor for DFS in patients with rectal cancer receiving nCRT and TME. The V30 of PBM was a useful predictive factor for the level of pre-SLR. Further study is warranted on preserving pre-SLR with bone marrow–sparing IMRT in patients with locally advanced rectal cancer.

## Data Availability

The data and materials in the current study are available from the corresponding author on reasonable request.

## Abbreviations

nCRT: Neoadjuvant concurrent chemoradiotherapy
ALCs: Absolute lymphocyte counts
pre-SLR: Pre-SL to pre-RTL ratio
HR: Hazard ratio
CI: Confidence interval
PBM: Pelvic bone marrow
DFS: Disease-free survival
OS: Overall survival
CEA: Carcino-embryonic antigen
TME: Total mesorectal excision
CTV: Clinical target volume
PTV: Planning target volume
